# Editorial: Transcriptome analysis in tumor microenvironment and tumor heterogeneity

**DOI:** 10.32604/or.2023.045719

**Published:** 2023-11-15

**Authors:** JINHUI LIU, JIAHENG XIE, PEIXIN DONG

**Affiliations:** 1 Department of Gynecology, The First Affiliated Hospital of Nanjing Medical University, Nanjing, 210029, China; 2 Department of Plastic and Cosmetic Surgery, Xiangya Hospital, Central South University, Changsha, 410008, China; 3 Department of Obstetrics and Gynecology, Hokkaido University School of Medicine, Hokkaido University, Sapporo, 060-8638, Japan

Cancer, a disease as intricate as it is devastating, continues to challenge the medical and scientific community [1]. Its complex nature is epitomized by the tumor microenvironment and tumor heterogeneity. As we delve deeper into the realms of cancer research, the advent of transcriptome analysis has emerged as a powerful torchbearer, illuminating our understanding of these enigmatic facets of cancer biology [2].

The tumor microenvironment (TME) has emerged as a pivotal player in cancer progression, response to therapy, and overall patient prognosis. Comprising a dynamic interplay of immune cells, stromal cells, blood vessels, and extracellular matrix components, the TME creates a nurturing niche for tumor cells to thrive [3]. Transcriptome analysis now provides us with a panoramic view of the intricate dialogues between these diverse cellular constituents.

By scrutinizing the RNA profiles of the TME’s inhabitants, researchers are deciphering the symphony of gene expression patterns that orchestrate the fate of tumors [4]. Signaling pathways that drive immune evasion, angiogenesis, and metastasis are being exposed, offering a treasure trove of therapeutic targets. Armed with this knowledge, we are better poised to develop interventions that disrupt the supportive TME, weakening the fortress that cancer constructs.

Within the heart of each tumor resides a mosaic of cellular diversity known as tumor heterogeneity. This phenomenon underpins treatment resistance, relapse, and therapeutic failures. Herein lies the challenge—treating a tumor as a monolithic entity ignores the multifaceted populations within. However, this challenge is met head-on by transcriptome analysis techniques [5].

Through RNA sequencing at single-cell resolution, we are able to dissect tumors into their cellular components, akin to peering through a microscope at a bustling metropolis. The unique gene expression profiles of distinct subpopulations unveil their characteristics, vulnerabilities, and interactions. Armed with this molecular cartography, researchers can navigate the labyrinth of tumor heterogeneity, directing therapies to target specific subclones that would otherwise escape attention.

This special issue in Oncology Research, entitled “Transcriptome Analysis in Tumor Microenvironment and Tumor Heterogeneity,” is presented by three guest editors. As of August 30, 2023, a total of four original studies have been published. Among them, Bao et al. conducted an insightful study where they employed transcriptome analysis to construct a solute carrier-associated prognostic signature for renal clear cell carcinoma [6]. This pioneering research has significant implications for the clinical management of patients with this form of kidney cancer. The development of this prognostic signature marks a critical advancement in personalized medicine for renal clear cell carcinoma patients. By stratifying patients based on their risk profiles, this signature offers clinicians a powerful tool to tailor treatment strategies, ensuring that each individual receives the most appropriate and effective care. One intriguing aspect of their findings is the identification of sensitive drugs for different subgroups of patients. This is particularly noteworthy because it not only allows for more targeted therapies but also hints at the potential for improved treatment outcomes and reduced side effects. It would be fascinating to delve deeper into the specific drugs recommended for each subgroup and explore the underlying mechanisms that make them particularly effective. Furthermore, this research underscores the growing importance of transcriptome analysis in oncology. It would be interesting to discuss how this approach can be applied to other types of cancer and whether similar prognostic signatures can be developed for different malignancies. The groundbreaking work conducted by Gu et al. sheds light on the intricate mechanisms underlying the promotion of gastric cancer progression, specifically through the role of apolipoprotein C1 (APOC1), as elucidated through multi-omics analysis [7]. This study not only advances our understanding of gastric cancer biology but also opens up promising avenues for diagnosis and immunotherapeutic interventions. One of the remarkable contributions of this research is the comprehensive expression map of APOC1 across various cancer types. This map not only provides valuable insights into the role of APOC1 in gastric cancer but also offers a broader perspective on its involvement in other malignancies. Liu et al.’s investigation into the heterogeneity of PD-L1 expression in non-small cell lung cancer represents a significant advancement in our understanding of the potential therapeutic implications of this immune checkpoint protein [8]. Their comprehensive approach, combining transcriptome analysis of the Cancer Genome Atlas (TCGA) with immunohistochemical staining, provides valuable insights into the intricate landscape of PD-L1 expression in lung cancer. The work conducted by Shi et al. represents a significant breakthrough in ovarian cancer research, with implications that extend to the development of more precise and effective therapeutic approaches [9]. Their approach, which combines single-cell sequencing analysis and the establishment of a superior prognostic model, sheds light on a novel target for ovarian cancer, CD38. One of the key aspects of this study is the identification of CD38 as a novel therapeutic target for ovarian cancer. CD38’s role in cancer progression and its potential as a target for therapy may open up new avenues for drug development and treatment strategies. It would be interesting to discuss the mechanisms through which CD38 contributes to ovarian cancer and how targeting it could lead to more effective therapies. The establishment of a superior prognostic model based on the T-cell depletion gene is particularly noteworthy. Such models are vital tools for predicting patient outcomes and tailoring treatments accordingly. It would be valuable to delve into the specific genes and factors incorporated into this model and how they correlate with ovarian cancer prognosis.

In summary, our special issue focuses on using transcriptome analysis to decipher tumor microenvironment and tumor heterogeneity and provide meaningful targets and prognostic models for them. In the era of precision medicine, understanding the intimate intricacies of cancer is no longer a distant dream. Transcriptome analysis stands as the bridge between our curiosity and comprehension, enabling us to decode the conversations within the tumor microenvironment and decipher the hieroglyphics of tumor heterogeneity. As we stand on the cusp of revolutionary advancements, let us celebrate the insights gleaned from these transcriptomic revelations, for they hold the key to unlocking the mysteries that cancer has guarded for far too long. Armed with this knowledge, we are equipped to forge innovative treatments that confront cancer’s complexity with unwavering precision.

## Data Availability

Not applicable.
